# Aggregation and Its Influence on the Bioactivities of a Novel Antimicrobial Peptide, Temporin-PF, and Its Analogues

**DOI:** 10.3390/ijms22094509

**Published:** 2021-04-26

**Authors:** Yu Zai, Xinping Xi, Zhuming Ye, Chengbang Ma, Mei Zhou, Xiaoling Chen, Shirley W. I. Siu, Tianbao Chen, Lei Wang, Hang Fai Kwok

**Affiliations:** 1 Institute of Translational Medicine, Faculty of Health Sciences, University of Macau, Avenida da Univesidade, Taipa, Macau, China; yuzai@um.edu.mo; 2 School of Pharmacy, Queen’s University Belfast, 97 Lisburn Road, Belfast BT9 7BL, UK; zye04@qub.ac.uk (Z.Y.); c.ma@qub.ac.uk (C.M.); m.zhou@qub.ac.uk (M.Z.); x.chen@qub.ac.uk (X.C.); t.chen@qub.ac.uk (T.C.); l.wang@qub.ac.uk (L.W.); 3 Jiangsu Key Laboratory of Biofunctional Molecule, College of Life Sciences and Chemistry, Jiangsu Second Normal University, Nanjing 210013, China; 4 Department of Computer and Information Science, Faculty of Science and Technology, University of Macau, Avenida da Universidade, Taipa, Macau, China; shirleysiu@um.edu.mo

**Keywords:** temporin, peptide aggregation, MD simulation, antimicrobial activity

## Abstract

Temporin is an antimicrobial peptide (AMP) family discovered in the skin secretion of ranid frog that has become a promising alternative for conventional antibiotic therapy. Herein, a novel temporin peptide, Temporin-PF (TPF), was successfully identified from *Pelophylax fukienensis*. It exhibited potent activity against Gram-positive bacteria, but no effect on Gram-negative bacteria. Additionally, TPF exhibited aggregation effects in different solutions. Three analogs were further designed to study the relationship between the aggregation patterns and bioactivities, and the MD simulation was performed for revealing the pattern of the peptide assembly. As the results showed, all peptides were able to aggregate in the standard culture media and salt solutions, especially CaCl_2_ and MgCl_2_ buffers, where the aggregation was affected by the concentration of the salts. MD simulation reported that all peptides were able to form oligomers. The parent peptide assembly depended on the hydrophobic interaction via the residues in the middle domain of the sequence. However, the substitution of Trp/D-Trp resulted in an enhanced inter-peptide interaction in the zipper-like domain and eliminated overall biological activities. Our study suggested that introducing aromaticity at the zipper-like domain for temporin may not improve the bioactivities, which might be related to the formation of aggregates via the inter-peptide contacts at the zipper-like motif domain, and it could reduce the binding affinity to the lipid membrane of microorganisms.

## 1. Introduction

Amphibian skin secretion is an outstanding source for discovering novel bioactive compounds that demonstrate potential for the development of new drugs in the treatment of human diseases [1,2,3]. Like the antimicrobial peptides (AMPs) from other sources (e.g., plant, insect, and mammal), amphibian skin-derived AMPs usually consist of 8–48 amino acid residues, generally possess at least one positive charge, and exhibit an amphipathic conformation [4]. Those AMPs have been considered to build the first line of innate immunity against the pathogenic microorganisms in their habitat [5]. They have also been proven to facilitate the absorption of skin-derived toxins to protect them from predators, as AMPs can permeabilize their epithelial tissue to enable fast transmembrane transport for co-secreted toxins [6]. So far, there are more than 1200 AMPs that have been identified through the skin secretions of different species (DRAMP database v2.0) [7]. They have generally been classified as Aurein [8], Dermaseptin [9], Phyllseptin [10], Phylloxin [11], and Medusin [12], which are found in Hylidae tree frogs; Brevinin [13], Esculentin [14], Japonicin [15], Nigrocin [16], Palustrin [17], Ranatuerin [18], Ranacyclin [19], and Temporin [20], which are discovered from Ranidae frogs; Kassinatuerin [21], which is isolated from genus *Kassina*; and Magainin [22], which is discovered from the genus *Xenopus*.

**Figure 1:**
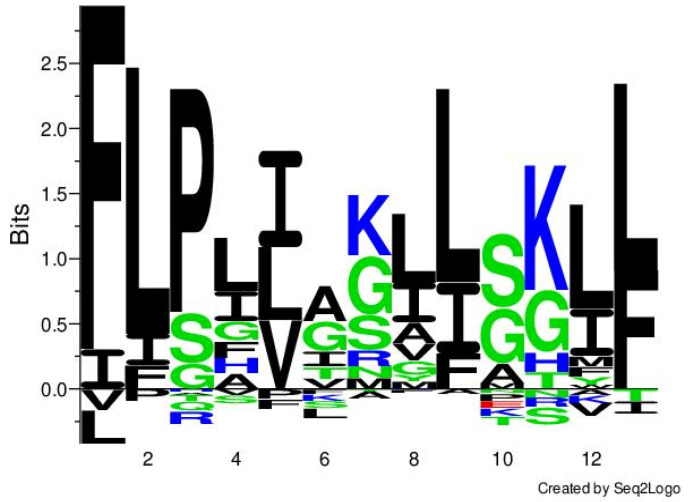
The sequence cluster of selected temporins from the DRAMP 2.0 database. The bits indicated the frequency of the presence of each amino acid residue in the position. The analysis was achieved by Seq2Logo online software. The black, green, blue, and red color represent hydrophobic, hydrophilic, basic, and acidic amino acid residues, respectively.

Among these antimicrobial peptide families, temporins comprise a group of relatively short peptides, widely distributed among ranid frogs [23]. The first member of Temporin was discovered from the skin secretion of European red frog *Rana temporaria* in 1996 [24]. They usually consist of 10–14 amino acid residues and contain net positive charges in a neutral environment [23]. Additionally, Temporins have demonstrated a high degree of structural similarity, while most demonstrate an N-terminal FLP motif and more than 50% hydrophobic amino acid residues constitution, such as phenylalanine (F), leucine (L), and isoleucine (I). They often possess one or two positive charges in the middle and the C-terminal regions, where the presence of lysine (K) is predominant (Figure 1). Temporins also contain an α-amidated C-terminus. They can adopt an α-helical structure in a membrane-mimicking environment [25].

Temporins often exhibited potent antimicrobial activity against the growth of Gram-positive bacteria, while the effect on Gram-negative bacteria is weaker [26]. The antimicrobial killing effect is generally recognized to permeabilize the cell membrane, resulting in the disruption of the structure [27]. Additionally, they also demonstrated potential in other aspects, including antifungal [28], anticancer [28], antiparasite [29], anti-inflammatory [30], and antiviral effects [31]. The native temporins show considerable drawbacks to the clinic application, such as significant hemolysis and cytotoxicity [32]. Moreover, forming oligomers in some microenvironments, such as lipopolysaccharides (LPS) of Gram-negative bacteria, could escalate the difficulty for temporin to traverse the outer membrane, which weakens the antimicrobial activity on Gram-negative bacteria [30,33]. Therefore, structural modifications and engineering have been continuously implemented for temporins to improve the therapeutic index and efficacy [20,26,30,34].

In this study, we discovered a novel temporin from the skin secretions of *Pelophylax fukienensis*, namely Temporin-PF (TPF: FLPLIAGLFGKIF-NH_2_), and observed that TPF tended to aggregate. Moreover, the peptide sequence presents a Phe zipper-like motif at its C-terminus, which could be associated with aggregation effects [35]. To investigate the relationship between the aggregation patterns and bioactivities, three analogs were synthesized with the removal of Phe and the substitution of Trp/D-Trp in the zipper-like motif domain. MD simulation was employed to elucidate the aggregation pattern among different analogs. In addition, the biofunctions of all peptides were evaluated via a series of biofunctional assays, including antimicrobial, antibiofilm, hemolysis, and the SYTOX green stain uptake assays.

## 2. Results

### 2.1. Identification and Characterization of Temporin-PF from the Skin Secretion

**Figure 2:**
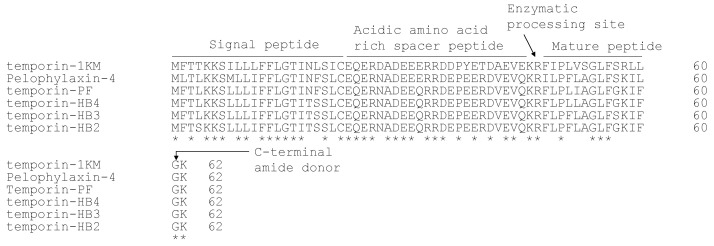
The alignment of translated open reading frame amino acid sequences of biosynthetic precursors. Temporin-1KM (accession No: AGE10577), Pelophylaxin-4 (accession No: Q2WCN5), and temporin-HB2,3,4 (accession No: AIU99904, AIU99906, AIU99905) were found in the skin secretion of *Pelophylax nigromaculatus*, *Pelophylax fukienensis*, and *Pelophylax hubeiensis*, respectively. The topological structure of precursors is indicated by a single line and arrows. The same amino acids of six peptides are marked with asterisks.

The prepropeptide encoding cDNA was successfully and consistently cloned from the skin-derived cDNA library (Supplementary Figure S1). The translated open-reading frame consists of 62 amino-acid residues, and it contains five topological domains. As shown in Figure 2, a signal peptide domain consists of 22 amino acid residues and is terminated by a cysteine residue. After the signal peptide domain, there is an acidic amino acid-rich spacer peptide domain that contains a large proportion of Asp and Glu residues. A mature peptide domain is followed by a typical di-basic peptide convertase processing site (-KR-) at the C-terminal region of the peptide precursor. The mature peptide in the skin secretion and the presence of the C-terminal amide were further confirmed by LC-MS/MS (Supplementary Figure S2), which is consistent with amide donor, Gly, at the end of the biosynthetic precursor. Through the BLAST analysis, the biosynthetic precursor exhibited a high degree of similarity to the temporin family members. The alignment with the top five hits identified from the skin secretion of *Pelophylax* species demonstrates the highly conserved signal peptide domain and spacer peptide domain. At the same time, their mature peptides also share several common features, including a Pro at position 3 (of mature peptide), a basic residue at position 11, and a large proportion of the hydrophobic amino acid residues (Figure 2). The mature peptide identified from the skin secretion was named temporin-PF (TPF), and the nucleotide sequence of cDNA has been deposited in the Genbank database under an accession number: MT663742.

### 2.2. Peptide Synthesis and Characterization

**Figure 3:**
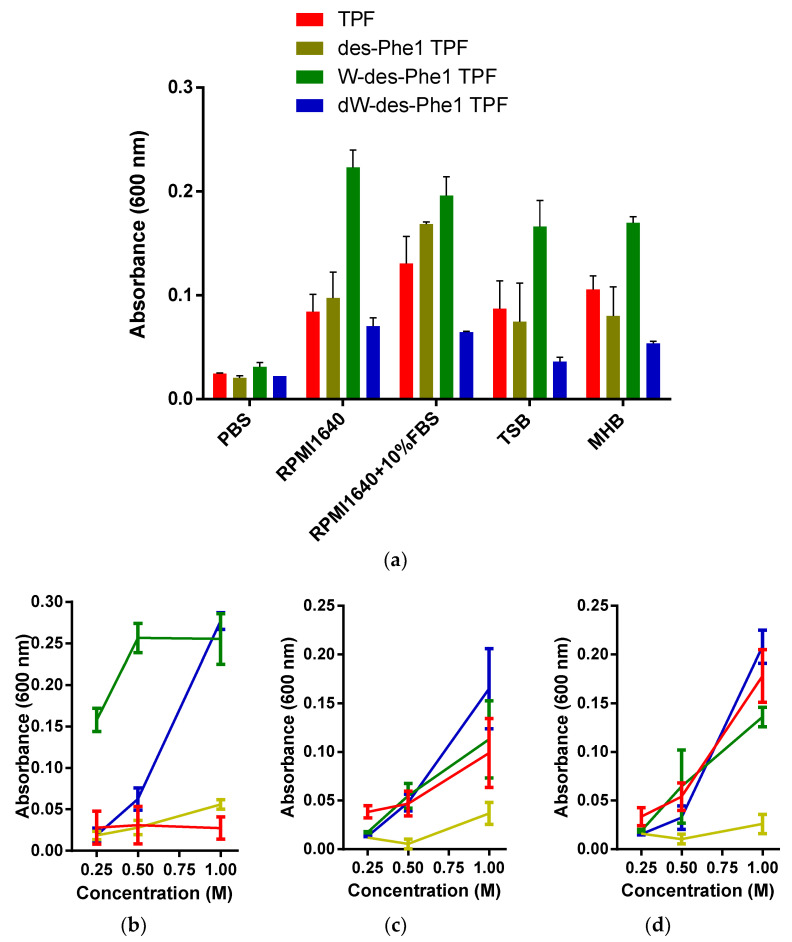
The absorbance of TPF and the analogs prepared in (**a**) a series of culture media, including PBS, RPMI1640, RPMI1640 with 10% FBS, TSB, and MHB; 0.25, 0.5, and 1 M of (**b**) Na_2_HPO_4_, (**c**) CaCl_2_, and (**d**) MgCl_2_ solutions. Peptide solutions were prepared from 1 mg/mL, and the wavelength was set as 600 nm. The error bar represents the standard deviation (SD) with three replicates.

After the identification of TPF, the chemical synthesis of TPF was performed successfully. Interestingly, the preliminary observation of TPF showed that the solubility of the peptide (10 mg/mL) was significantly decreased in PBS (Figure 3). It formed hydrogel-like morphology at room temperature within a short period (a couple of minutes) (Supplementary Figure S3). As a previous study showed, temporin tends to oligomerize with residues assembly from the N- and C-termini, where the N-terminal Phe binds to C-terminal Leu through hydrophobic interaction [36]. Therefore, we synthesized the des-Phe1 TPF to investigate the correlation between oligomerize effect and bioactivities. Moreover, TPF demonstrated a phenylalanine zipper motif at the C-terminus, which has been suggested to facilitate the assembly of temporin L [35]. Therefore, we further substituted Phe with Trp and D-Trp to reveal the relationship between the oligomerization effect and the zipper motif and the influence on bioactivities (Table 1). The correct synthesis of peptides had been checked by MALDI-TOF mass spectrometry (Supplementary Figure S6) and synthetic yields were calculated (Supplementary Figure S7).

### 2.3. Observation of Aggregation Effect of Synthetic Peptides

All peptides were chemically synthesized and purified by RP-HPLC. They were further mixed with an equal ratio and subjected to RP-HPLC. The order of retention time for all analogs is TPF> des-Phe1 TPF> W-des-Phe1 TPF> dW-des-Phe1 TPF (Supplementary Figure S4). Subsequently, all peptides (1 mg/mL) were dissolved in a series of widely used culture media (Figure 2). All peptides exhibited different aggregation patterns. Specifically, the turbidity of W-des-Phe1 TPF was increased outstandingly among four culture media. Furthermore, all peptides demonstrated “salting-out” effects in, respectively, 0.25, 0.5, and 1 M of Na_2_HPO_4_, CaCl_2_, or MgCl_2_ solutions. In the Na_2_HPO_4_ solutions, W-des-Phe1 TPF consistently exhibited the strongest salting-out effect among the four peptides, while in the other three solutions, TPF, W-des-Phe1 TPF, and dW-des-Phe1, TPF showed a similar effect. des-Phe1 TPF displayed a low degree of capability for aggregation in salt solutions.

### 2.4. MD Simulation of Aggregation Effect of Peptides

**Figure 4:**
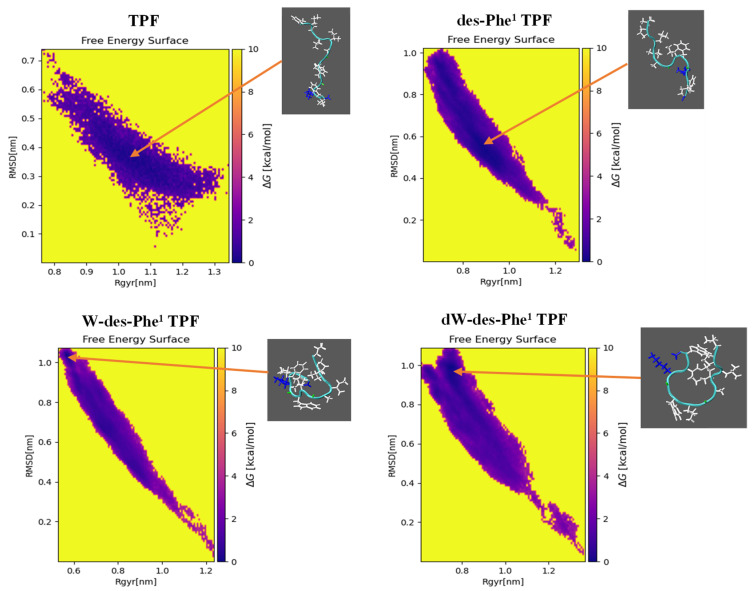
The plot of the free energy of each MD trajectory frame of the folding of TPF and the analogs for 100 ns simulation in a water box, in terms of RMSD and Rgyr from a linear extended structure. Arrows indicate the represented frames with the lowest free energy.

To reveal the aggregation patterns of TPF and analogs, MD simulations were employed. The initial monomolecular simulation for each peptide was conducted in a water box for 100 ns with a modified water model, TIP4P-D, and Amber99SB-ILDN forcefield. They can substantially reproduce the experimental observables for intrinsically disordered proteins and peptides [37,38]. The energy analysis demonstrated that each peptide monomer system was stable and validated, as seen in Supplementary Figure S5. The peptide with the lowest free energy was found to display a random coil conformation (Figure 4) and selected for the following aggregation simulation through cluster analysis.

**Figure 5:**
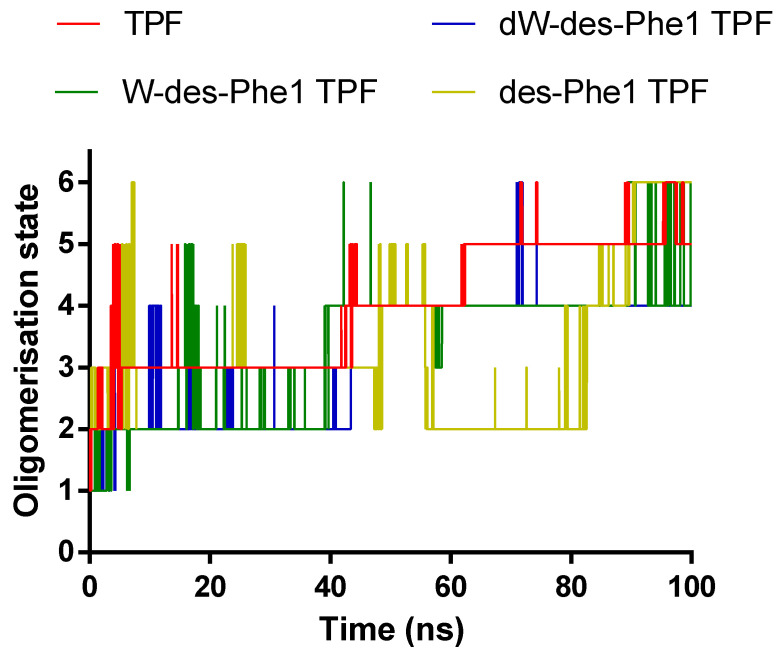
The changes of oligomerization state of TPF and the analogs during the 100-ns MD simulation. Each line represents the maximum oligomer size in the system.

**Figure 6:**
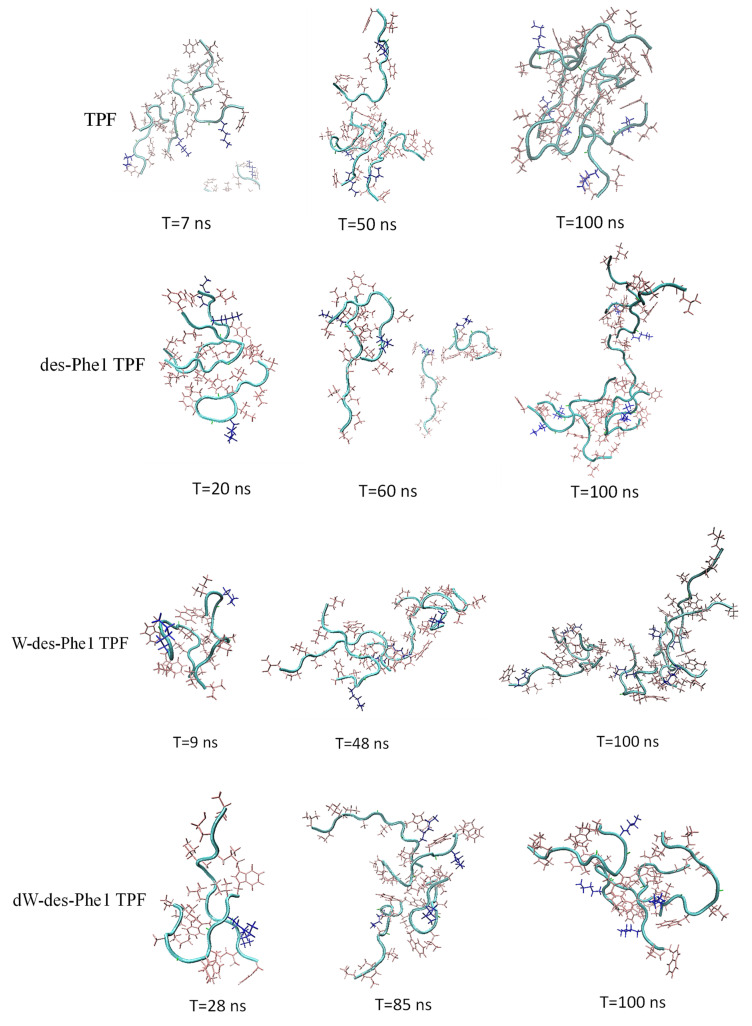
The snapshots of the trajectory of the aggregation simulation of the peptides. The frames at different time points were represented based on the oligomerization state analysis in Figure 5. The positively charged residues and nonpolar residues were represented by blue and pink, respectively.

**Figure 7:**
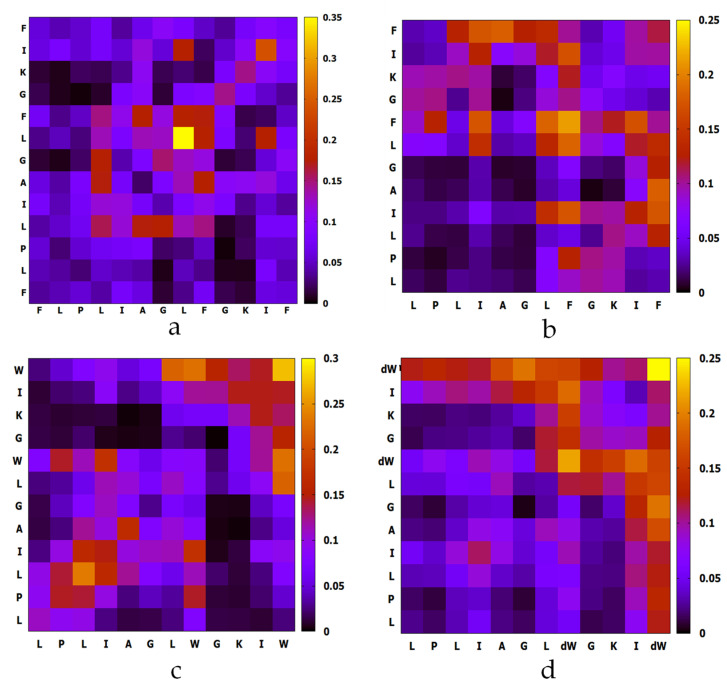
The contact heat maps of the amino acid residues between any of two monomers of (**a**) TPF, (**b**) des-Phe1 TPF, (**c**) W-des-Phe1 TPF, and (**d**) dW-des-Phe1 TPF in the formation of an oligomer during the 100 ns simulation aggregation of six peptide monomers. The color scale indicates the probabilities of contact between any of the two peptide monomers.

For MD simulation of aggregation, six peptide molecules were randomly inserted in a water box. The molecules were defined to form an oligomer once any of two atoms from either molecule were within a minimum distance of less than 0.4 nm. The MD trajectories revealed that TPF and analogs were able to oligomerize within a short time. As Figure 5 shows, all peptides were able to form dimer or trimer at the beginning of inter-peptide interaction, and the size of the oligomer continuously increased, except for des-Phe1 TPF, which demonstrated dissociation and reassociation events from 40 to 100 ns. As Figure 6 showed, TPF tended to form a pentamer at the end of the simulation, while des-Phe1 TPF and W-des-Phe1 TPF possessed a dynamic transition between tetramer and hexamer. dW-des-Phe1 TPF formed a stable tetramer in the 100 ns simulation. The snapshots of the aggregation trajectory revealed that TPF was able to form a stable trimer in the early stage of the simulation (Figure 6). Consistent with the data shown in the inter-residue contact heat maps (Figure 7), TPF assembled through the hydrophobic interaction with hydrophobic residues in the middle domain. In contrast, des-Phe1 TPF showed enhanced packing effect via the C-terminal Phe zipper motif. When substituting the two Phe residues with Trp and D-Trp, stronger contacts in this region were observed.

### 2.5. Secondary Structure Analysis of TPF and the Analogs

**Figure 8:**
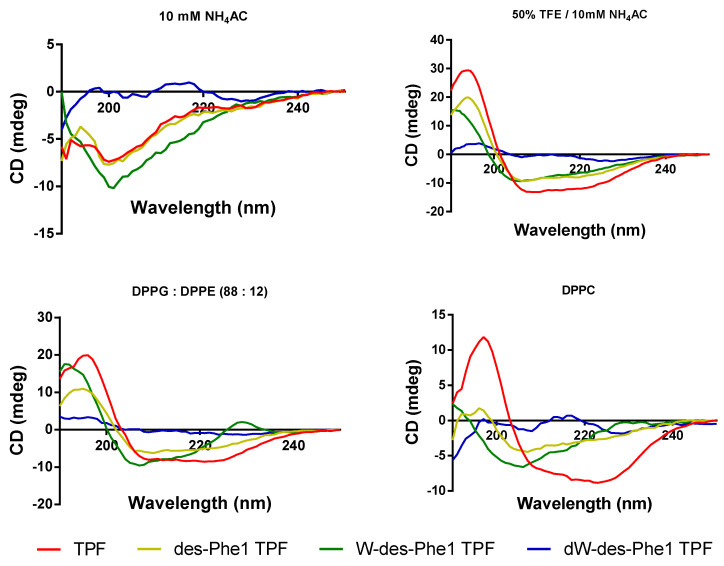
CD spectra of TPF and the analogs (50 μM) prepared in 10 mM NH_4_AC, 50% TFE/10 mM NH_4_AC, 3 mM DPPG/DPPE (88:12) SUVs, and 3 mM DPPC SUVs. The spectra were averaged over three consecutive scans, and the solvent CD signal was subtracted.

The CD analysis reveals that TPF and the analogs, except dW-des-Phe1 TPF, produced α-helical conformations in 50% TFE/10 mM NH_4_AC with two negative bands in the CD spectra centered at 208 and 222 nm and a positive band centered at 193 nm (Figure 8). In comparison, TPF and the analogs exhibited slightly negative ellipticity around 222 nm in 10 mM ammonium acetate solution, indicating all peptides formed random coil structures in the aqueous environment. These data are consistent with the MD simulations for all peptides. DPPG:DPPE (88:12) and DPPC small unilamellar vesicles (SUVs) were used to mimic the bacterial and human erythrocyte membranes, respectively. TPF, des-Phe1 TPF, and W-des-Phe1 TPF can adopt a helical structure in both membrane-mimicking environments. However, TPF exhibited a deeper peak at 222 nm, indicating that its secondary structure contains a higher proportion of helical structure. Table 2 shows that the helical contents of TPF, des-Phe1 TPF, and W-des-Phe1 TPF decrease in DPPC SUVs compared to DPPG:DPPE (88:12) SUVs, indicating that the peptides may exert selectivity towards the bacterial cell membrane. Additionally, dW-des-Phe1 TPF did not present a helical conformation in any of the environment.

### 2.6. Antimicrobial, Antibiofilm, and Hemolytic Activity of TPE and the Analogs

**Figure 9:**
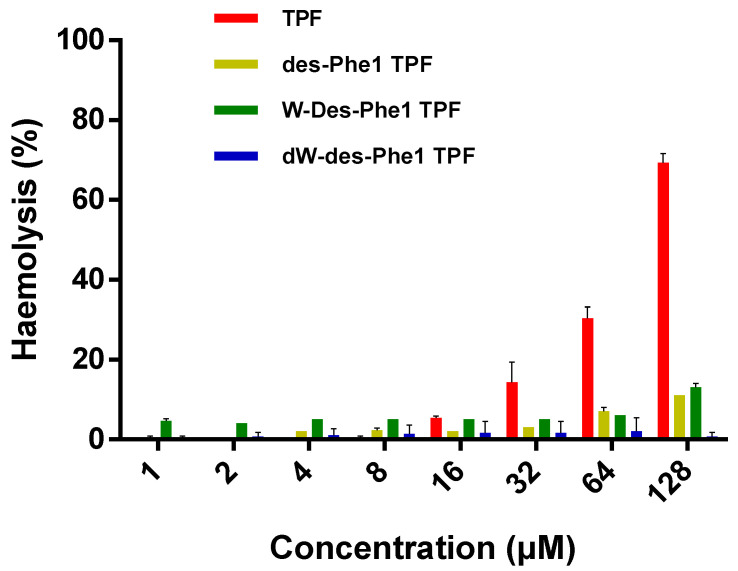
Hemolytic activity of TPF and the analog against horse red blood cell. The percentage of hemolysis was calculated with the absorbance at 570 nm, compared with PBS-treated red blood cells. The error bar represents SD of five replicates.

TPF exhibited antimicrobial activity against selected Gram-positive bacteria, while it was ineffective against Gram-negative strains at a concentration up to 128 µM. The three analogs revealed an overall weaker effect than the parent peptide. However, des-Phe1 TPF showed a slightly increased effect against the growth of *E. coli* (Table 3). Additionally, TPF and des-Phe1 TPF not only inhibited the growth of bacterial biofilm, but also destroyed the mature bacterial biofilm of both MRSA and *S. aureus*, while W-des-Phe1 TPF only showed a weak antibiofilm effect. dW-des-Phe1 TPF did not possess any antibiofilm activity at the highest concentration (Table 4). Similarly, TPF possessed the strongest hemhemolytic activity against horse erythrocytes, while des-Phe1 TPF and W-des-Phe1 TPF exerted less than 20% hemhemolysis at the concentration up to 128 µM. dW-des-Phe1 TPF demonstrated negligible effect compared with the other peptides (Figure 9).

### 2.7. S. aureus Membrane Permeabilization

**Figure 10:**
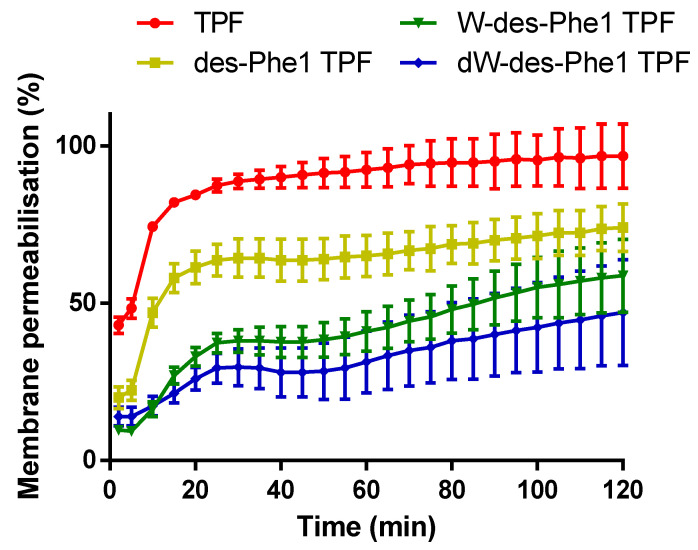
Membrane permeabilization effect of TPF and the analogs (10 µM) on Gram-positive bacteria *S. aureus* was indicated by the uptake of SYTOX Green dye over 120 min. The error bar represents SD of three replicates.

As a complementary analysis of the antimicrobial function, the ability of TPF and the analogs (at 10 µM) to compromise the cell integrity of *S. aureus* were also examined (Figure 10). TPF permeabilized the cell membrane of *S. aureus* and induced the uptake of SYTOX dye remarkably. It gave rise to around 50% membrane permeabilization within a few minutes. des-Phe1 TPF showed a lower effect compared with the parent peptide, while it still resulted in over 50% membrane permeabilization within 120 min. However, W-des-Phe1 TPF and dW-des-Phe1 TPF exhibited similar and weak membrane-compromising effect on *S. aureus* cells.

## 3. Discussion

As described in previous studies, Temporins, which exert antimicrobial activity, LPS neutralization effect, and aggregation properties, have been considered to be promising lead compounds for drug discovery [29,35,39]. Herein, we discovered a novel temporin member, TPF, and synthesized three analogs to investigate the relationship among bioactivities, aggregation patterns, and amino acid substitutions.

Interestingly, TPF and the analogs were able to form a hydrogel in PBS, while they were “salting-out” in the other buffers, especially with the presence of di-cationic ions. It suggested that the aggregation potencies of the peptides depend on the concentration and the type of the ions and solutes present. Additionally, the turbidity of the peptide solution increased when using the cell and bacteria culturing media, indicating that the peptide could precipitate via aggregation with large molecules, like proteins and peptides, in the culturing media. N-terminal Phe of temporin was believed to participate in the packaging of “head-tail” oligomerization [30,33]. Therefore, as the MD simulation data showed, the aggregation effect was decreased when removing the N-terminal Phe from TPF. Furthermore, Trp/D-Trp substitution carried out on des-Phe1 TPF enhanced the peptide aggregation. As the MD simulation reported, W-des-Phe1 TPF and dW-des-Phe1 TPF demonstrated enhanced inter-peptide interaction through the zipper motif at the C-terminus, which could result in strong aggregation property. Moreover, the experimental results of antimicrobial activity showed that the activity of the analogs had been significantly reduced. It may be related to the decrease of the helical content; however, it could also be caused by the change of aggregation effects. Previous studies have shown that AMPs with strong antimicrobial activity are present as monomers in an aqueous solution and aggregate on the membrane to disrupt the bacterial cell membrane [40,41]. However, when the aggregation ability is too strong, they may start to aggregate before attaching on the cell membrane, suggesting that their potency to disrupt the cell membrane could be affected and reduced [42,43]. It has been proposed that some aggregation effects of AMPs could limit the interaction of the peptide with its target, thereby reducing the antibacterial efficacy [44,45]. In the previous study, the zipper-like motif is associated with the bioactivities as well as the assembly in the LPS micelles, which was considered to arise from hydrophobic interactions mainly [35]. However, the overall hydrophobicity of Trp/D-Trp substituted analogs is less than that of des-Phe1 TPF, as estimated by RP-HPLC analysis. We hypothesize that the enhanced assembly seen for Trp/D-Trp substituted analogs might be related to the increased content of aromatic residues, because it could promote the strength of π stacking or cation-π type interaction [46]. However, the true relation has not been fully clarified so far, and the capability of aromatic moieties to promote aggregation could also be affected by the hydrophobicity, position, and the neighboring side chains in their three-dimensional conformation.

Temporins usually induce potent antimicrobial activity and considerable hemolytic activity [20,23,34]. Collectively, a high proportion of lipophilic, hydrophobic, and amphipathic structure content tends to result in both antimicrobial potency and hemolysis [32,47]. In this study, the native TPF showed activity against *S. aureus* only, while the removal of ^1^Phe improved the effect on *E. coli* slightly as well as decreased activity against *S. aureus*. As previous studies have reported, the N-terminal Phe promoted the packaging of “head–tail” oligomerization [30,33]. This conformation is effective in disruption of lipid bilayers, but it could confer restricted translocation through the cell wall/outer membrane of bacteria. As Gram-negative bacteria contain an LPS outer membrane, the oligmerized peptides have a weaker diffusion effect than the monomer to penetrate the outer membrane. It might also explain that the weak aggregation property of des-Phe1 TPF would allow more efficient translocation from the LPS outer membrane [33], so that more peptide molecules could interact with the plasma cell membrane. Due to the removal of Phe, the antimicrobial activity was decreased, either induced by the loss of the head-tail packaging, or simply caused by the decrease of hydrophobicity or the reduced helical content. Additionally, the other two analogs showed even weaker bioactivities, suggesting that inappropriate aggregation could reduce the membrane permeabilization effect.

In terms of decreased hemolysis of the analogs, the decrease of overall hydrophobicity would protect the cell membrane of erythrocytes from non-specific disruption. On the other hand, as Table 2 suggested, all peptides exhibited lipid selectivity, since they adopted a higher conformational propensity for helical structure in bacterial cell membrane mimicking SUVs (DPPG:DPPE) than in mammalian membrane mimicking SUVs (DPPC), because the helical structure could be stabilized by charge–charge interaction [48]. Moreover, a helical conformation is vital for AMPs to exert the membrane permeabilization effect. Usually, a large proportion of helical content could result in significant enhancement on the formation of transmembrane pores [49]. Therefore, the stronger membrane permeabilization effect of TFP could be associated with its high helical content. Interestingly, it is noticeable that the CD spectra of TFP are different between DPPG: DPPE and DPPC SUVs, which cannot be explained at this stage. It is hypothesized that some AMPs may present in different aggregation patterns from partitioning to self-assembly with different lipid constitutions [50].

The MBIC and MBEC values are similar to their respective MIC and MBC, suggesting the antibiofilm mechanisms may be similar to the bacteria-killing mechanisms. Especially, the MBIC being equal to MIC, indicates that the peptides simply kill the most bacteria so that there would be less viable cells to produce biofilm. Regarding the eradication concentration, the removal of mature biofilm would arise from a detergent-like function. Therefore, a strong membrane permeabilization effect would contribute to a potent antibiofilm activity, which seems to be unlikely to involve the interference with quorum sensing and bacterial communication during the biofilm formation.

Tryptophan is a commonly occurring amino acid in antimicrobial peptides, and it plays an important role in exerting the antibacterial effect, and in previous studies, it was also found that the aromatic residues were critical for broad-spectrum activity [51,52]. Moreover, Trp residues confer a high propensity to interact with the interfacial region of bacterial cell membranes, and Trp-containing peptide may kill Gram-negative bacteria by a “self-promoted uptake” pathway [53]. However, the SYTOX green dye uptake assay showed that the Trp substituted analogs did not fully permeabilize the cell membrane of *S. aureus* to the same degree as the parent, which most likely arises from the strong self-assembly effects that eliminated the pore formation procedures in the cell membrane. On the other hand, we also observed that TPF exhibited the strongest biological activity with a high oligomerization state, indicating that the aggregation pattern of temporin is essential for exerting bioactivities. Although our MD simulation did not represent the head–tail dimer as shown previously [36], TPF showed fewer possibilities on assembly via the Phe zipper motif that may ensure the capability to bind to the lipid membrane through hydrophobic interaction with the C-terminal domain.

## 4. Materials and Methods

### 4.1. Specimen Biodata and Harvesting of Skin Secretion

Specimens of *Pelophylax fukienensis* (*n* = 3, snout-to-vent length 7 cm) were captured in Fuzhou City, Fujian Province, China. All frogs were adults, and skin secretion was obtained by a mild electrical stimulation on the dorsal skin surface of the frogs, as outlined previously [12]. The secretion was collected by washing the skin using deionized water and was lyophilized after the liquid nitrogen freezing. The study was performed according to the guidelines in the UK Animal (Scientific Procedures) Act 1986, project license PPL 2694, issued by the Department of Health, Social Services, and Public Safety, Northern Ireland. Procedures had been vetted by the IACUC of Queen’s University Belfast, and approved on 1 March 2011.

### 4.2. Identification of Precursor-Encoding cDNA from the Skin Secretion

The precursor encoding cDNA from the skin secretion was obtained as described previously. Briefly, mRNA in the skin secretion was isolated and reverse-transcribed to the cDNA. A degenerate sense primer (5′-GAWYYAYYHRAGCCYAAADATG-3′) that was designed according to the highly conserved 5′-untranslated region of temporin encoding mRNA from ranid frogs was employed to conduct 3′-RACE reaction to obtain the full-length open reading frame. The PCR transcripts were purified and cloned using a pGEM-T vector system (Promega Corporation, Madison, WI, USA) and sequenced using an ABI 3700 automated sequencer (Applied Biosystems, Foster City, CA, USA). The expression of mature peptide in the skin secretion was further confirmed by LC-MS/MS analysis using Thermo LCQ Fleet mass spectrometer as outlined previously [54].

### 4.3. Chemical Peptide Synthesis

Fmoc solid peptide synthesis chemistry was implemented using an automatic peptide synthesizer (Protein Technologies, Inc., Tucson, AZ, USA), as described in the previous study [12]. Briefly, the Fmoc protected amino acids were deprotected by 20% piperidine. Then, the released α-NH_2_ was coupled with carboxy group in the next amino acid with the presence of HBTU and 1 M NMM. The Rink amide MBHA resin was employed to provide the C-terminal amide for each peptide. Then, the peptide chain was removed from resin by a reaction cocktail including TFA, EDT, thioanisole, and water (94/2/2/2; *v*/*v*/*v*/*v*). The peptide was precipitated by diethyl ether and further purified by RP-HPLC with a preparative column (C18, 21.2 × 250 mm), using a standard gradient of acetonitrile/water mobile phases. Matrix-assisted laser desorption ionization and time-of-flight mass spectrometry (MALDI-TOF MS) (Voyager DE, Perspective Biosystems, Foster City, CA, USA) were applied to check the correct synthesis of the peptide.

### 4.4. Observation of Aggregation of The Peptides

The method was followed the previous study with modifications [43]. The purified peptides were prepared at 10 mg/mL in water and subsequently diluted with different solutions, including PBS, RPMI1640, RPMI1640 with 10% FBS, TSB, MHB, Na_2_HPO_4_ (0.25, 0.5, and 1 M), CaCl_2_ (0.25, 0.5, and 1 M), and MgCl_2_ (0.25, 0.5, and 1 M), in a 96-well plate to achieve the final concentration of 1 mg/mL. The turbidity of each well was detected using the absorbance at 600 nm by a plate reader. The peptides dissolved in water were applied as negative control.

### 4.5. Molecular Dynamic Simulation

The MD simulation was conducted using GROMACS 2020.4 package [55]. Each peptide monomer was built using UCSF Chimera [56] and further represented by the protein forcefield Amber99SB-ILDN [38] in combination with the modified water model TIP4P-D. The solvated water box was defined as a 7.5 nm cubic box with 0.15 M of sodium and chlorine ions to neutralize the net charge of the system and ensure the physiological salinity of the solution. Each peptide system was energy minimized by the steepest descent with a maximum force tolerance of 100 kJ/mol/nm and equilibrated for 100 ps with position restrain simulations at 298 K with a coupling constant of 0.1 ps and Berendsen coupling algorithm at 1 bar with a coupling constant of 1 ps. The MD simulation for each peptide monomer was performed by a Leap-Frog integrator with a time-step of 2 fs for 100 ns, where constraints included all bonds using the LINCS algorithm.

Once the MD simulation for monomer was achieved, the energy analysis was conducted using GROMACS tools. The free energy surface analysis of each peptide monomer was performed by a python script generateFES.py (http://www.strodel.info/index_files/lecture/generateFES.py, accessed on 20 December 2020), employing the RMSD of the Cα of the peptide from each frame and radius of gyration of the peptide. The peptide conformation with the lowest free energy value was selected for simulation of peptide aggregation. The method followed the previous study [57] for investigating the aggregation of six peptide molecules that were randomly inserted in a 10 nm cubic water box in combination with a Charmm36-mar2019 forcefield. The energy minimization was performed similarly with a maximum force tolerance of 500 kJ/mol/nm. The first quick equilibration for 100 ps was performed where the temperature was increased to 300 K using v-rescale thermostat with a coupling constant of 0.1 ps. The second equilibration for 2 ns was implemented where the pressure of the system was further equilibrated at 1 bar using the Parrinello–Rahman algorithm with a coupling constant of 2 ps. The production simulation was performed similarly as above, with a time-step of 2 fs for 100 ns. The oligomerization state and the contact map of residues were analyzed using the python script analysis.py (https://github.com/strodel-group/Oligomerization-State_and_Contact-Map, accessed on 25 January 2021).

### 4.6. Circular Dichorism

The determination of the secondary structure of peptides was conducted using a JASCO J-815 CD Spectropolarimeter (JASCO Inc., Tokyo, Japan) as described in the previous study [54]. Then, 50 μM of each peptide sample was prepared in the aqueous 10 mM ammonium acetate (NH_4_Ac; Sigma-Aldrich, Gillingham, UK) buffer, 50% (*v*/*v*) 2,2,2-trifluoroethanol (TFE; Sigma-Aldrich, Gillingham, UK), and 3 mM DPPG:DPPE (88:12) and DPPC small unilamellar liposomes/vesicles (SUVs) that were used to mimic the bacterial and mammalian erythrocyte cell membranes, respectively. The liposome was hydrated in deionized water and then sonicated for 15 min with an interval 30 s in an ice bath for generating SUVs. Samples were loaded in a 1-mm thickness quartz cuvette and analyzed at room temperature with the following parameters: scan range of 190–250 nm, scanning speed of 100 mm/min, 1 nm bandwidth, and 0.5 nm data pitch. The spectra were obtained by averaging data from three scans. The CD spectra were analyzed by BeStSel (http://bestsel.elte.hu/index.php, accessed on 16 May 2020) for the determination of secondary structure content.

### 4.7. Antimicrobial Assays

The minimum inhibitory concentration (MIC) and minimum bactericidal concentration (MBC) of peptides against the selected microorganisms were determined using the broth-dilution method, as described in the previous study [12]. Seven different microorganisms were used in the assay: Gram-positive bacteria *Staphylococcus aureus* (NCTC 10788), *Enterococcus faecalis* (NCTC 12697), and *methicillin-resistant S. aureus* (*MRSA*) (NCTC 12493); Gram-negative bacteria *Escherichia coli* (NCTC 10418), *Pseudomonas aeruginosa* (ATCC 27853), and *Klebsiella pneumoniae* (ATCC 43816). All the strains were cultured in Mueller Hinton Broth (MHB), pH 7.4 (Oxiod, Basingstoke, UK). Each peptide was tested at the final concentration from 128 to 1 µM in two-fold dilution. Norfloxacin was applied as the positive control.

### 4.8. Determination of Minimal Biofilm Inhibitory Concentration (MBIC) and Minimal Biofilm Eradication Concentration (MBEC)

MBIC and MBEC assays were performed as previously described with minor modifications [15]. For the MBIC assay, a suspension of broth diluted bacteria culture (5 × 10^5^ CFU/mL) was incubated with the peptide solutions at 37 °C for 24 h. For MBEC assay, 100 μL inoculum culture was seeded to a 96-well-flat-bottom plate and incubated at 37 °C for 48 h to obtain the mature biofilm. Afterwards, the plate was washed by sterile phosphate-buffered saline (PBS; Sigma-Aldrich, Gillingham, UK) twice and treated with a series peptide solution at 37 °C for 24 h. Then, all the plates were washed with PBS and stained by 100 μL 0.1% crystal violet solution (Sigma-Aldrich, Gillingham, UK), and further dissolved by 30% acetic acid (Sigma-Aldrich, Gillingham, UK). The absorbance of each well was recorded by Synergy HT plate reader (Biotech, Minneapolis, MN, USA) at 595 nm.

### 4.9. Hemolysis Test

The hemolytic activity of each peptide was measured by incubating a range of final peptide concentrations from 128 to 1 µM in a two-fold dilution in a 2% suspension of the horse erythrocytes, as described in a previous study [54]. The absorbance of hemoglobin released from red blood cells was detected at 570 nm. The percentage of hemolysis was calculated by the comparison to the 1% Triton X-100 treated red blood cells.

### 4.10. SYTOX Green Stain Uptake Assay

A membrane permeability assay was conducted on *S. aureus* (NCTC 10788) and *E.coli* (NCTC 10418) using the SYTOX™ Green Nucleic Acid Stain (Thermo Fisher Scientific, Waltham, MA, USA) as described previously, with some modifications. For *S. aureus*, to obtain the fluorescence kinetics of membrane permeabilization, the bacterial suspension and peptide solutions, prepared in 5% tryptic soy broth (TSB) (*v*/*v*) in 0.85% NaCl solution (*m*/*v*), were mixed to give a final concentration at 10 µM in a black 96-well plate, and further incubated for 120 min at 37 °C. Subsequently, 5 µM SYTOX Green nucleic acid stain was added into each well and immediately detected by a fluorescent plate reader with excitation of 485 nm and emission of 528 nm. The total permeabilized cells of *S. aureus* were prepared by treating with 70% isopropanol and further resuspended in 5% TSB/0.85% NaCl solution after washing.

## 5. Conclusions

In summary, this study discovered a novel temporin peptide from skin secretion and revealed its aggregation pattern by “salting out” assays and MD simulation. The precision of the simulation may vary based on the forcefield and the defined water molecules. Therefore, we employed circular dichroism tests to validate the secondary structure that was computed by MD simulation. Successfully, consistent results were observed from both approaches, ensuring the confidence of the peptide conformation. However, some flaws such as the unobservable reported head to tail binding feature of temporin were contained in the simulation. Therefore, the higher-order peptide structure may also need to be tested by using NMR to validate the tertiary and quaternary structures and the exposure of the residue side chain on the molecule surface. For the aggregation analysis, our data suggested that the N-terminal Phe of TPF plays a key role in the self-assembly in the solution. It seems that loss of Phe1 resulted in the disordered packaging via the zipper-like domain, and enhancing aromaticity in such domain would change the assembly pattern and facilitate undesirable aggregation effect. Therefore, introduction of Trp for improving antimicrobial activity may deliver a counterintuitive effect, which may be caused by promoting the aggregation effect through π stacking or cation-π type interaction. Although these hypotheses need to be further validated by NMR analysis, our study can bring structural insight into the rational design of temporins with the enhanced antimicrobial activity and proper peptide oligomerization.

## Figures and Tables

**Table 1:** Primary sequences of Temporin-PF and its analogs.

Peptide	Sequence	Length (Residues)	Mono Molecular Weight (Da)	Charge
TPF	FLPLIAGLFGKIF-NH_2_	13	1433.88	2
des-Phe1 TPF	LPLIAGLFGKIF-NH_2_	12	1286.81	2
W-des-Phe1 TPF	LPLIAGLWGKIW-NH_2_	12	1364.83	2
dW-des-Phe1 TPF	LPLIAGLWGKIW-NH_2_	12	1364.83	2

The bold typeface represents the D-form enantiomer.

**Table 2:** Helix content (%) in 50% TFE/10 mM NH_4_AC, 3 mM DPPG/DPPE (88:12), and DPPC analyzed by BeStSel (http://bestsel.elte.hu/index.php).

Peptide	Helix Content %		
	50% TFE	DPPG/DPPE	DPPC
TPF	58.9	41.4	27.7
des-Phe1 TPF	37.7	26.7	16.4
W-des-Phe1 TPF	27.7	26.9	10.1
dW-des-Phe1 TPF	3.0	0.5	8.3

**Table 3:** The minimal inhibitory concentrations (MIC) and minimal bactericide concentrations (MBC) of Temporin-PF and the synthetic analog peptides against selected microorganisms.

Peptide	MIC/MBC (µM)					
	*S. aureus*	MRSA	*E. faecalis*	*E. coli*	*P. aeruginosa*	*K. pneumoniae*
TPF	4/32	4/4	16/16	>128/>128	>128/>128	>128/>128
des-Phe1 TPF	16/16	32/32	32/32	64/128	>128/>128	128/128
W-des-Phe1 0TPF	32/32	128/128	128/128	128/128	>128/>128	128/128
dW-des-Phe1 TPF	64/64	>128/>128	>128/>128	>128/>128	>128/>128	>128/>128

**Table 4:** The minimal biofilm inhibitory concentrations (MBIC) and minimal biofilm eradication concentrations (MBEC) of Temporin-PF and the synthetic analog peptides against *S. aureus* and *MRSA*.

Peptide	MBIC/MBEC (µM)	
	*S. aureus*	MRSA
TPF	4/32	4/16
des-Phe1 TPF	16/32	32/32
W-des-Phe1 TPF	64/>128	128/>128
dW-des-Phe1 TPF	>128/>128	>128/>128

## Data Availability

Not applicable.

## Supplementary Materials

The following are available online at https://www.mdpi.com/article/10.3390/ijms22094509/s1.
