# Supplementary material for: Aggregation and Its Influence on the Bioactivities of a Novel Antimicrobial Peptide, Temporin-PF, and Its Analogues
Source: Int J Mol Sci. 2021 Apr 26;22(9):4509. doi: 10.3390/ijms22094509 (PMC8123395; doi:10.3390/ijms22094509)
Supplement: Supplementary file 1 [file ijms-22-04509-s001.zip › ijms-1170643-supplementary.pdf]

```
   M  F  T  L   K  K  S   L  L  L   I  F  F  L   G  T  I ·
  1ATGTTCACCT TGAAGAAATC CCTGTTACTC ATTTTCTTCC TTGGGACCAT
   TACAAGTGGA ACTTCTTTAG GGACAATGAG TAAAAGAAGG AACCCTGGTA
 · N  F  S   L  C  E  Q   E  R  N   A  D  E   E  Q  R  R ·
 51CAACTTTTCT CTCTGTGAGC AGGAGAGAAA TGCCGATGAG GAACAAAGAA
   GTTGAAAAGA GAGACACTCG TCCTCTCTTT ACGGCTACTC CTTGTTTCTT
 ·  D  E  P   E  E  R   D  V  E  V   Q  K  R   F  L  P
101GAGACGAGCC AGAGGAAAGA GATGTTGAAG TACAAAAACG TTTTCTACCA
   CTCTGCTCGG TCTCCTTTCT CTACAACTTC ATGTTTTTGC AAAAGATGGT
   L  I  A  G   L  F  G   K  I  F   G  K  *
151CTTATCGCAG GCCTGTTCGG TAAAATTTTC GGAAAATAAC CAAAAAATGT
   GAATAGCGTC CGGACAAGCC ATTTTAAAAG CCTTTTATTG GTTTTTTACA
201TGAAACTTTG GAAAAGAAAT TGGAAATCAT CGGATGTGGA ATATCATTTA
   ACTTTGAAAC CTTTTCTTTA ACCTTTAGTA GCCTACACCT TATAGTAAAT
251GCTAAATGCA CATCAGATGT CTTAAAAAAA AATAAATAAA ACTCTTTACG
   CGATTTACGT GTAGTCTACA GAATTTTTTT TTATTTATTT TGAGAAATGC
301TCAACTCTAA AAAAAAAAAA AAAAAAAAAA
```

Figure S1. Nucleotide sequence of the cDNA cloned from the skin secretion of *Pelophylax fukienensis* and its predicted peptide sequence. The putative signal peptide and temporin-PF (TPF) are indicated by single underline and double underlines, respectively.

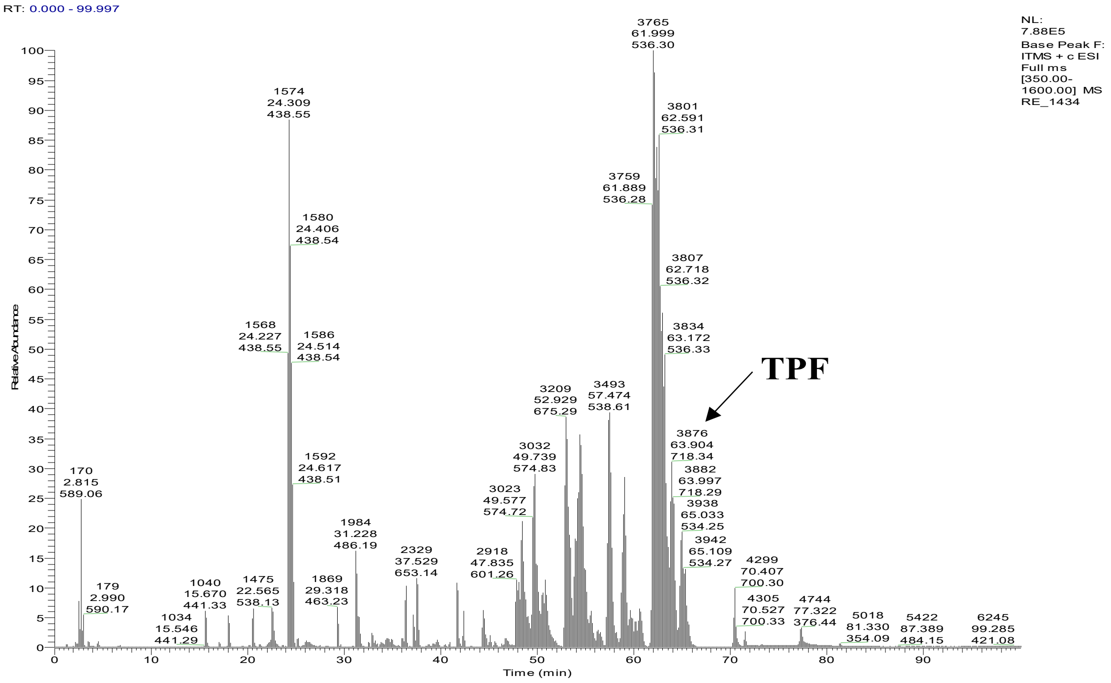

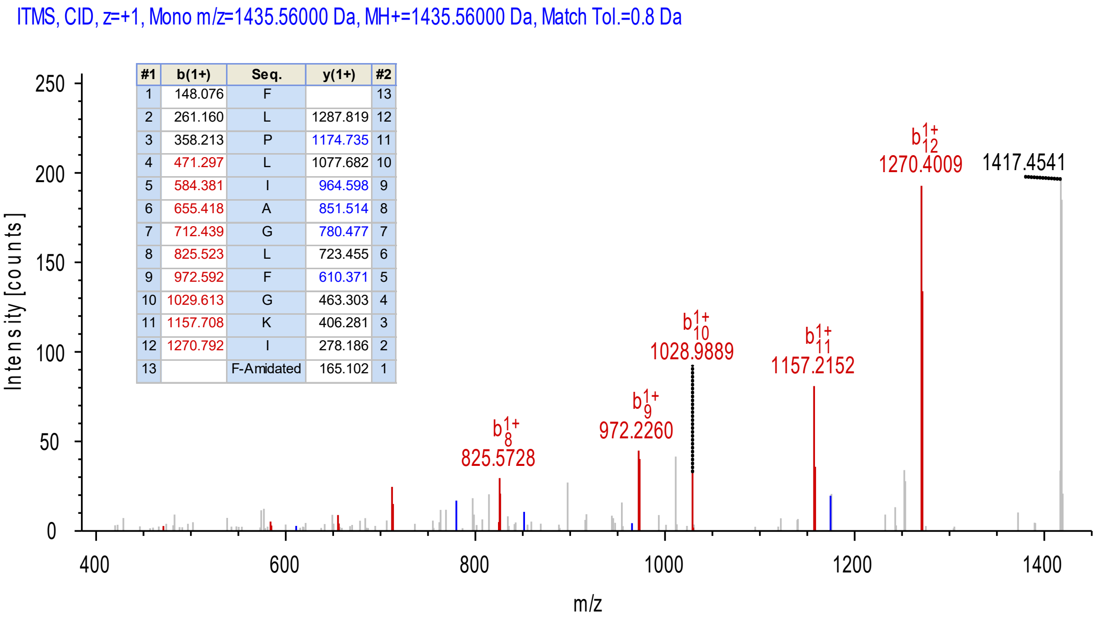

#1	b(1+)	Seq.	y(1+)	#2
1	148.076	F		13
2	261.160	L	1287.819	12
3	358.213	P	1174.735	11
4	471.297	L	1077.682	10
5	584.381	I	964.598	9
6	655.418	A	851.514	8
7	712.439	G	780.477	7
8	825.523	L	723.455	6
9	972.592	F	610.371	5
10	1029.613	G	463.303	4
11	1157.708	K	406.281	3
12	1270.792	I	278.186	2
13		F-Amidated	165.102	1

Figure S2. Identification of TPF derived from the skin secretion of *Pelophylax fukienensis*: (A) RP-HPLC chromatogram of skin secretion of *P. fukienensis* monitored at 214 nm. The arrow indicates the retention time of QUB-1434. (B) Annotated MS/MS spectrum of QUB-1434. Predicted b- and y-ions arising from collision induced dissociation of the mono-charged (1435.56 m/z, $[M+H]^+$) precursor ion. The observed b- and y-ions are indicated in red and blue typefaces.

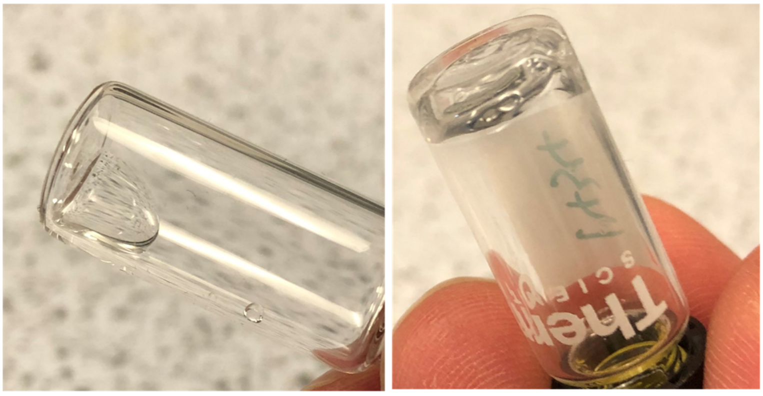

(**a**) (**b**)

Figure S3. The fluidity of (a) PBS and (b) 10 mg/ml TPF in PBS. The peptide is dissolved in PBS and formed hydrogel-like morphology.

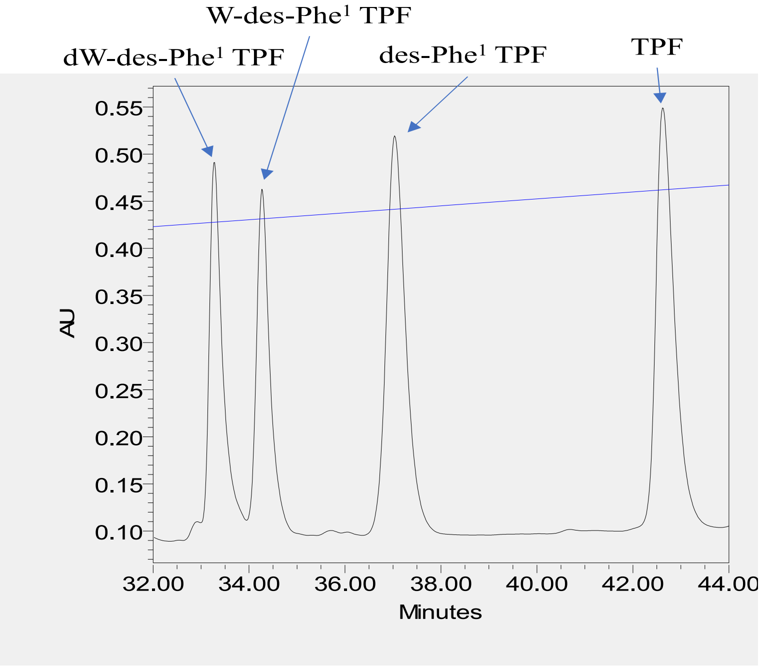

Figure S4. The chromatogram of the mixture of TPF analogues. The retention time of each peptide is indicated by arrow. The gradient of mobile phase is presented by a blue line.

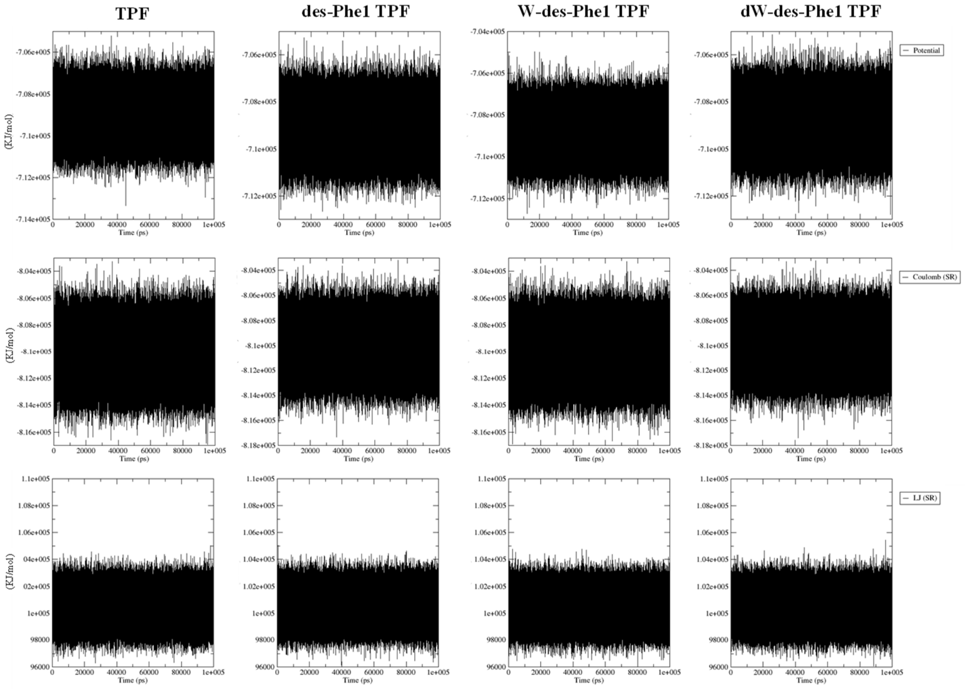

Figure S5. The energy analysis of TPF and the analogues monomer during 100 ns MD simulation.

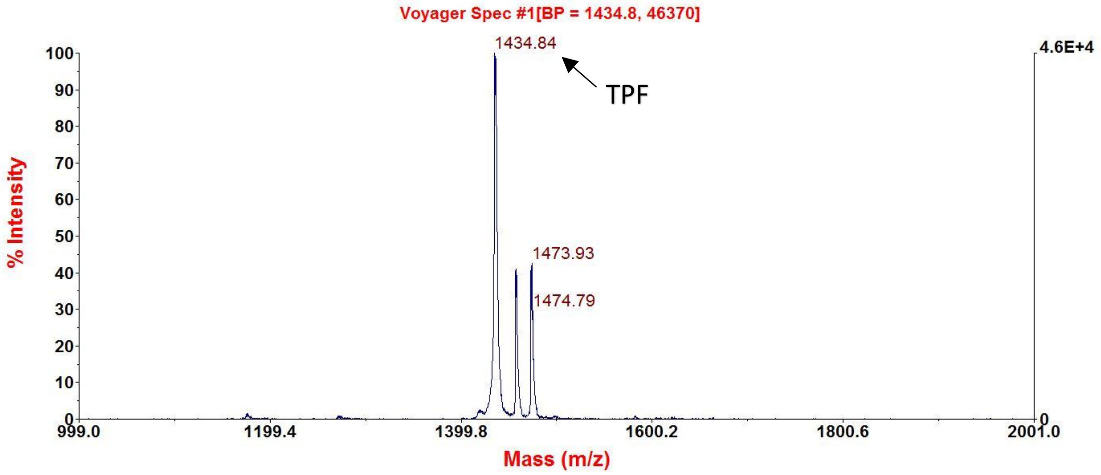
Voyager Spec #1[BP = 1434.8, 46370]
1434.84
TPF
1473.93
1474.79
% Intensity
Mass (m/z)
4.6E+4

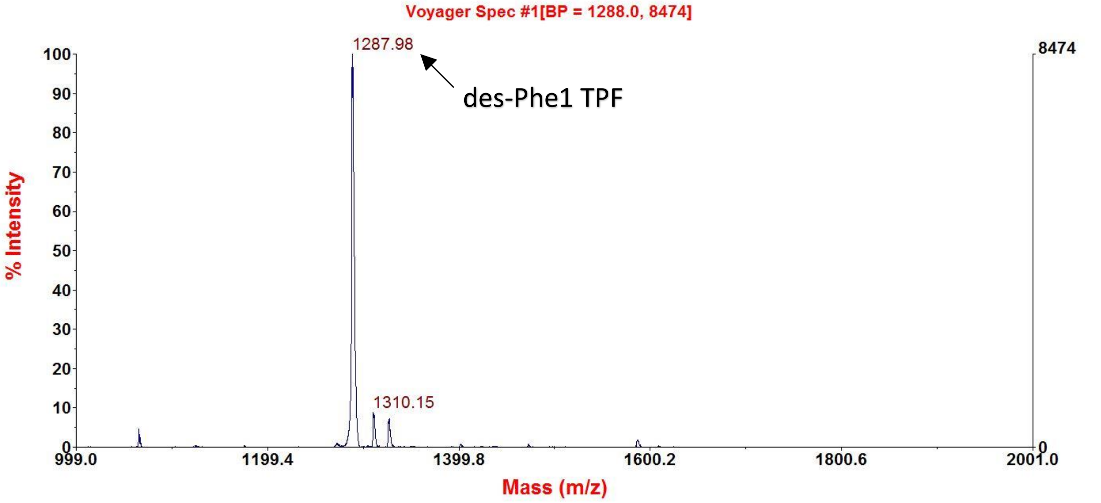
Voyager Spec #1[BP = 1288.0, 8474]
1287.98
des-Phe1 TPF
1310.15
% Intensity
Mass (m/z)
8474

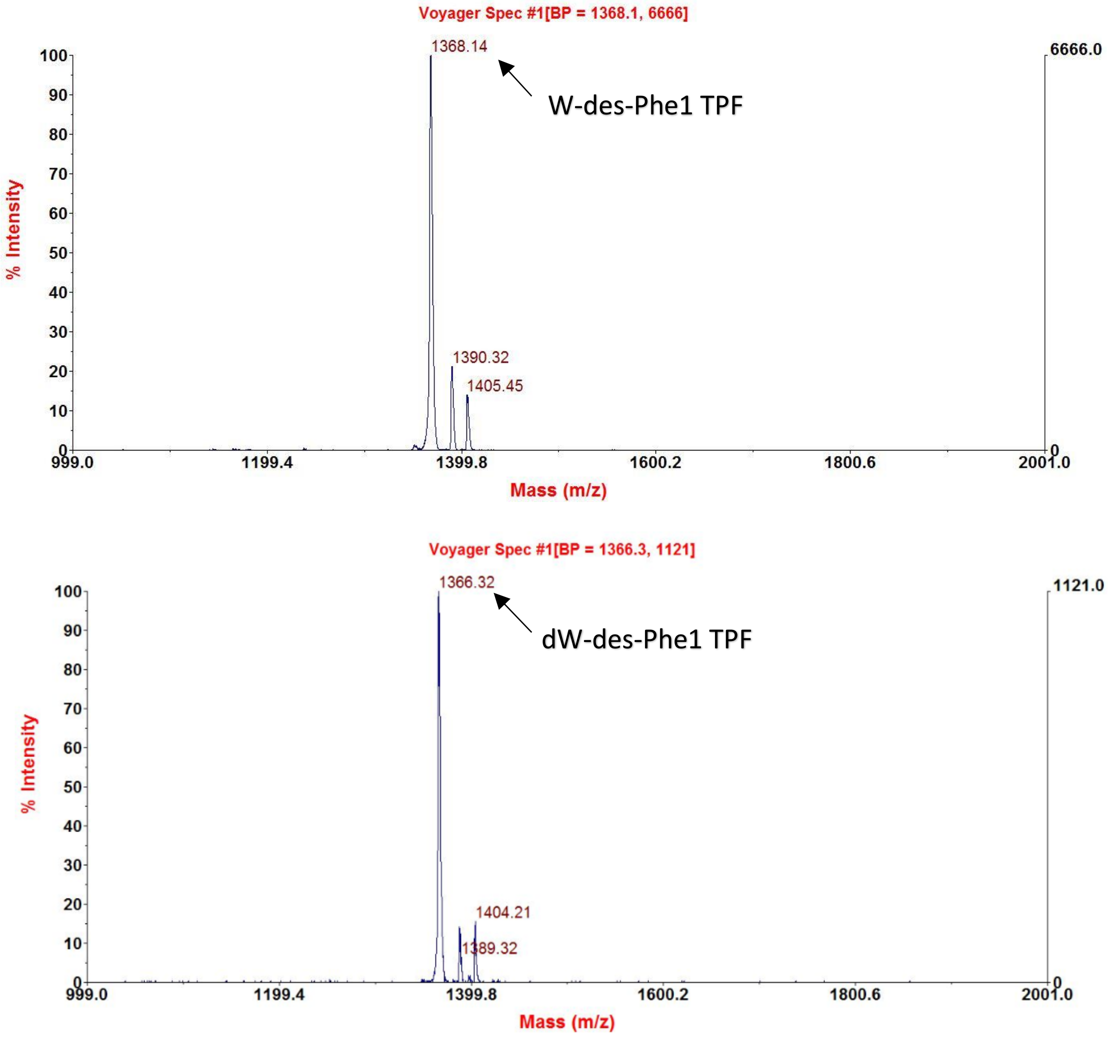

Figure S6. Mass of TPF and its analogues identified by matrix-assisted laser desorption ionization, time-of-flight mass spectrometry.

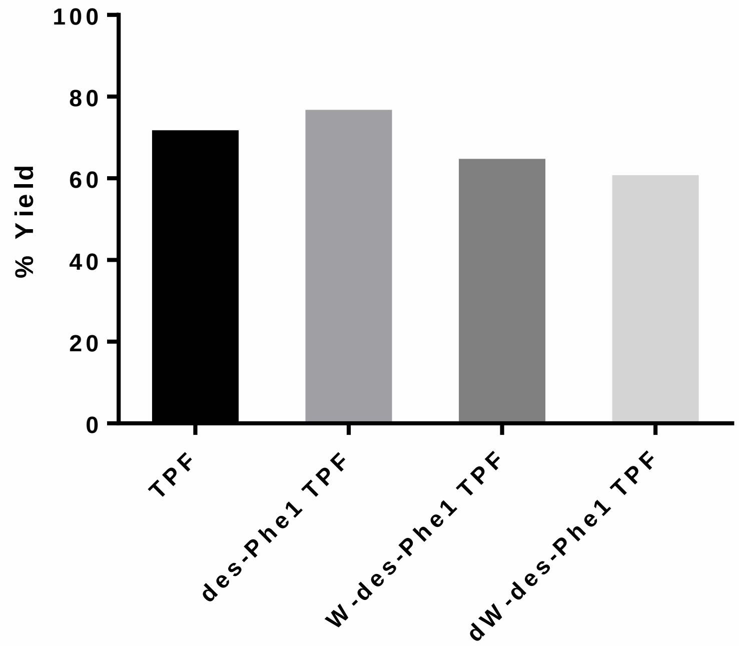

Figure S7. Synthetic yield of TPF and its analogues. The yield of TPF, des-Phe1 TPF, W-des-Phe1 TPF and dW-des-Phe1 TPF were 71%, 76%, 64% and 60%, respectively.
